# Anti-inflammatory properties of amniotic membrane patch following pericardiectomy for constrictive pericarditis

**DOI:** 10.1186/s13019-017-0567-7

**Published:** 2017-01-26

**Authors:** Katherine M. Marsh, Alice S. Ferng, Tia Pilikian, Ankit A. Desai, Ryan Avery, Mark Friedman, Isabel Oliva, Clint Jokerst, David Schipper, Zain Khalpey

**Affiliations:** 1 Department of Surgery, Division of Cardiothoracic Surgery, University of Arizona College of Medicine, 1501 North Campbell Avenue, Room 4302, Tucson, AZ 85724 USA; 2 Department of Internal Medicine, Division of Cardiology, University of Arizona College of Medicine, Tucson, USA; 3 Department of Medical Imaging, University of Arizona College of Medicine, Tucson, USA; 4 Department of Physiological Sciences, University of Arizona College of Medicine, Tucson, USA; 5 Department of Biomedical Engineering, University of Arizona College of Medicine, Tucson, USA; 6 Department of Translational and Regenerative Medicine, University of Arizona College of Medicine, Tucson, USA

**Keywords:** Constrictive pericarditis, Amniotic membrane patch, Orthotopic heart transplant

## Abstract

**Background:**

Since constrictive pericarditis is most often idiopathic and the pathophysiology remains largely unknown, both the diagnosis and the treatment can be challenging. However, by definition, inflammatory processes are central to this disease process. Amniotic membrane patches have been shown to possess anti-inflammatory properties and are believed to be immune privileged. Due to these properties, amniotic membrane patches were applied intraoperatively in a complicated patient presenting with constrictive pericarditis.

**Case presentation:**

A patient with a history of multiple cardiac surgeries presented with marked fatigue, worsening dyspnea and sinus tachycardia. He was found to have constrictive physiology during cardiac catheterization, with cardiac MRI demonstrating hepatic vein dilatation, atrial enlargement and ventricular narrowing. After amniotic membrane patch treatment and pericardiectomy, post-operative cardiac MRI failed to demonstrate any appreciable pericardial effusion or inflammation, with no increased T2 signal that would suggest edema.

**Conclusions:**

Given the positive results seen in this complex patient, we suggest continued research into the beneficial properties of amniotic membrane patches in cardiac surgery.

**Electronic supplementary material:**

The online version of this article (doi:10.1186/s13019-017-0567-7) contains supplementary material, which is available to authorized users.

## Background

Constrictive pericarditis results in a thickened and less-elastic pericardium, which can lead to incomplete diastolic filling and myocardial ischemia [1]. Since it is rare and the presenting symptoms are similar to those of several other disorders, the diagnosis can often be challenging. The diagnosis is usually made using cardiac catheterization or echocardiography as a part of the patient’s initial clinical evaluation. Although there are multiple etiologies of constrictive pericarditis, in most cases, the pathophysiology is idiopathic or may occur following cardiac surgical procedures including orthotopic heart transplant [2]. Constrictive pericarditis is commonly treated with pericardiectomy; however even following surgical intervention, long-term survival decreases over time and further diminishes when patient history includes multiple cardiac re-operations [3].

It has recently been demonstrated that amniotic stem cell therapy consisting of either stem cells with extracellular matrix or extracellular matrix alone can decrease fibrosis and post operative inflammation in humans [4, 5]. Specifically, extracellular matrix in the form of human amniotic membrane allograft has shown to significantly reduce post-ischemic cardiac dysfunction, improve ischemic heart repair, and increase blood flow recovery in rat and mouse models [6, 7]. This immunoprivileged tissue does not need to be donor-recipient matched to produce positive outcomes, further supporting its convenience of use and value [8]. In the context of these anti-inflammatory properties, and since inflammatory processes are central to the pathophysiology of pericarditis, amniotic membrane patches were applied intraoperatively in a patient presenting with constrictive pericarditis as outlined below.

## Case presentation

**Fig. 1:**
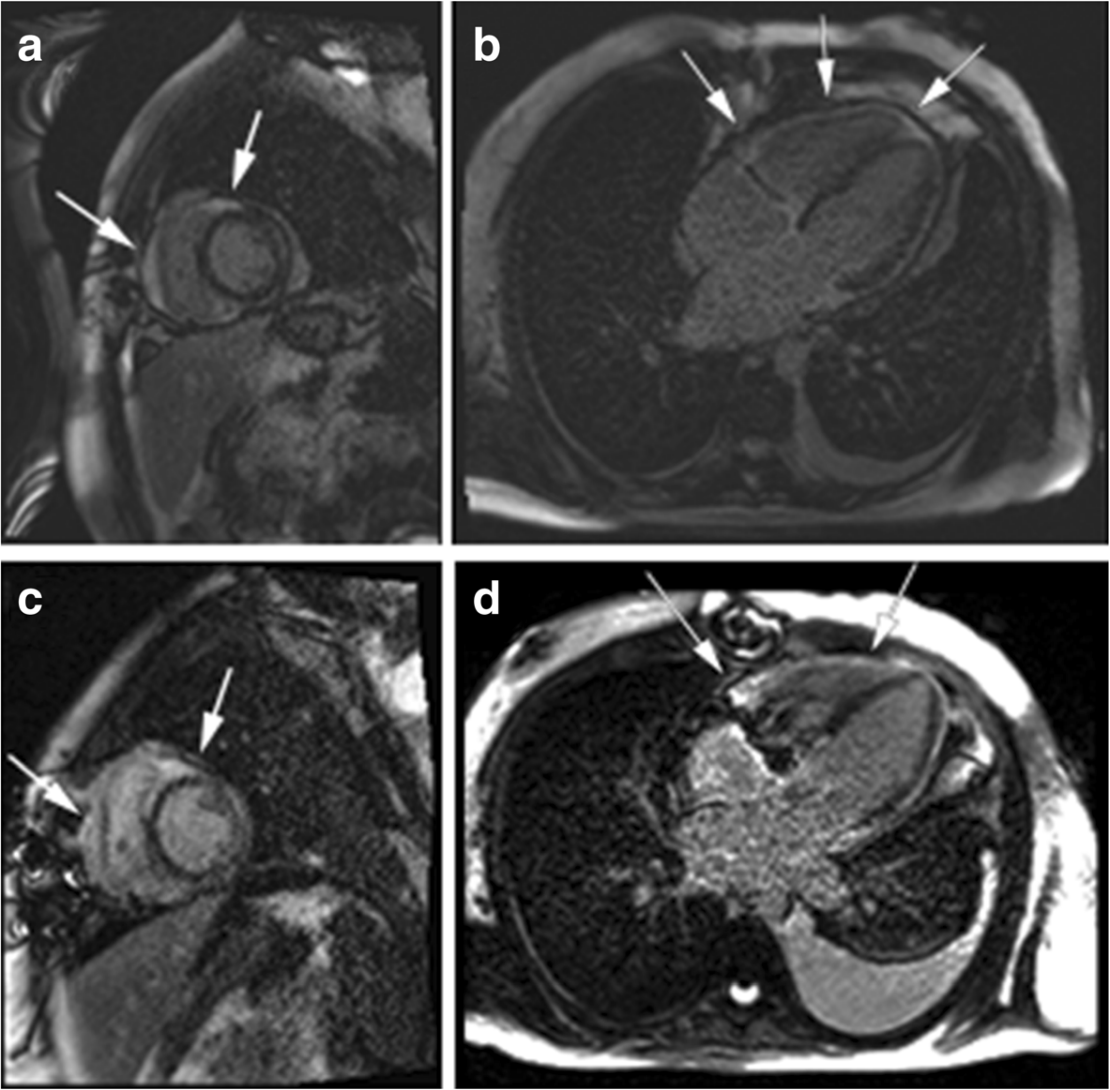
Post-contrast T1-weighted cardiac MRI images. Preoperative MRI short-axis (**a**) and 2-, 3-, and 4-chamber long axis (**b**) delayed-enhanced inversion-recovery images demonstrate pericardial thickening and epicardial enhancement (*arrows*) with subtle periventricular septal flattening. On dynamic cine imaging and anatomic imaging (not shown) there is septal bounce during Valsalva suggesting ventricular inter-dependance and biatrial enlargement with dilated IVC and hepatic veins. These findings are absent on postoperative cardiac MRI (**c**-**d**)

A 34-year-old male had a history of orthotopic heart transplant for hypertrophic obstructive cardiomyopathy and subsequent tricuspid repair for severe tricuspid regurgitation due to prolapse. The heart transplant was performed at a medical center in a neighboring state, with both prior surgeries performed by two different surgeons. He was doing well for over 3 years following these procedures, maintained with immunotherapy consisting of 2.5 mg Prograf® BID and 250 mg CellCept® daily as noted by the CT 7 Transplant Database. He then developed a one-week history of marked fatigue, worsening shortness of breath, and sinus tachycardia on electrocardiogram and was transferred to our facility from a neighboring state. Upon arrival, the patient’s ejection fraction was significantly reduced at less than 20%, compared to his usual baseline of 60% last recorded 1.5 years prior to this presentation. Given the concern for acute transplant rejection, the patient underwent endomyocardial biopsy via catheterization, which revealed evidence of Grade 1R mild acute cellular rejection without the presence of antibody-mediated rejection as evidenced by a pathologic antibody-mediated rejection (pAMR) of 0. Hemodynamic findings from right and left cardiac catheterization revealed equalization of diastolic filling pressures with discordance after volume loading, consistent with constrictive physiology. Additionally, Freidreich’s sign was present with both steep x and y descent of the jugular venous pressure (JVP) tracing with increased left and right ventricular diastolic pressures with dip and plateau (square root sign). The patient then underwent a cardiac magnetic resonance imaging (MRI) study to further evaluate the constrictive physiology. Cardiac MRI revealed mild pericardial thickening, mild flattening and bounce of the interventricular septum, intermediate epicardial signal on pre-contrast T2-weighted images and demonstrated intermediate delayed enhancement with post-contrast T1-weighted images consistent with fibrotic tissue (Fig. 1a, b). There was also moderate to moderate-severe tricuspid regurgitation. Given the hemodynamic values from cardiac catheterization and pericardial thickening, surgery was recommended to replace the tricuspid valve and remove the fibrotic epicardial material.

**Fig. 2:**
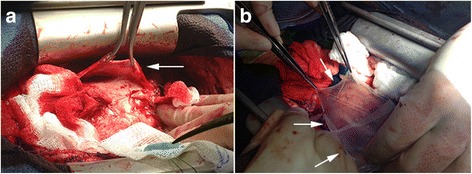
Intraoperative images. Thick gelatinous material was found on the anterior surface of the heart, with the pericardium and Gore-Tex membrane fused to the thickened pericardium at the anterior surface (**a**). Four human allograft membranes were topically placed over the right atrium, right ventricle, and left ventricle (**b**) for their anti-fibrotic and anti-inflammatory properties

**Fig. 3:**
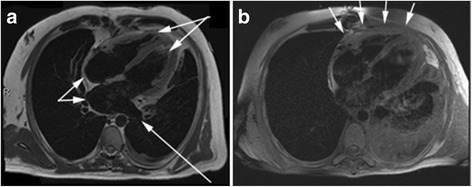
T2 Fat Sat Horizontal long-axis fat-suppressed T-2 weighted images. Preoperative cardiac MRI (**a**) reveals hepatic vein dilatation, atrial enlargement and ventricular narrowing (*arrows*). Post-operative cardiac MRI (**b**) fails to demonstrate any appreciable pericardial effusion or inflammation, with no increased T2 signal that would suggest edema (*arrows*)

Using a redo sternotomy approach, complete phrenic to phrenic pericardiectomy, removal of Gore-Tex membrane from previous surgery and a tricuspid valve replacement were completed. Gross intraoperative findings of the pericardial space included thick, gelatinous material on the anterior surface of the heart, and the pericardium and Gore-Tex membrane were fused to the thickened pericardium on the anterior surface of the heart. (Fig. 2a) Prior to closure, four human allograft membranes were topically placed over the right atrium, right ventricle and left ventricle (Fig. 2b) for their anti-fibrotic and anti-inflammatory properties [7, 8]. The surgical pathology report later confirmed fibrosis of the explanted tricuspid valve, pericardial fibrosis, and chronic pericardial inflammation. Though constrictive physiology was still noted, post-contrast T1-weighted cardiac MRI images demonstrate significant improvement (Fig. 1c, d). Furthermore, a five-week postoperative fat-suppressed T2-weighted MRI revealed no appreciable postoperative inflammation and an absence of pericardial effusion (Fig. 3).

## Discussion

Constrictive pericarditis is difficult to diagnose given its rare occurence and that the presenting symptoms are similar to those of several other disorders. In our patient, this diagnosis was further complicated by his history of orthotopic heart transplant. While the techniques continue to evolve, imaging patients in order to optimize diagnosis following cardiac transplant is limited [9]. Given our patient’s surgical history and clinical presentation, the constrictive physiology from cardiac catheterization was considered to be a more reliable diagnostic measure than both the MRI and echocardiogram findings. The case discussed serves as a prime example for the need to develop alternate non-invasive imaging techniques or algorithms for improved diagnostic capacity in patients with prior orthotopic heart transplant.

Another confounding variable in diagnosing constrictive pericarditis in our patient was the steep y descent on JVP. Although Freidreich’s sign is suggestive for constrictive pericarditis, our patient was also diagnosed with severe tricuspid regurgitation. However, despite these diagnostic difficulties, the patient was known to have prior Gore-Tex membrane placement during surgery. This iatrogenic component is another factor that may, in part, explain his constrictive physiology.

As discussed previously, pericardiectomy is often performed as a curative procedure for constrictive pericarditis. However there are instances, particularly for patients with advanced constrictive pericarditis or with radiation disease, in which pericardiectomy may not offer a cure or desired long-term result [1]. For these patients with higher risk factors, it may be beneficial to explore additional or alternative treatment options. Current treatment of recurrent pericarditis has focused on targeting inflammation, and has shown overall positive outcomes [3]. Given our patient’s extensive cardiac history of orthotopic heart transplant, reoperation for tricuspid valve repair, and Gore-Tex adhesions, extraordinary care was initiated in an attempt to resolve his constrictive pericarditis. As an emerging anti-inflammatory and anti-fibrotic treatment, the use of human allograft membrane has proven to be both safe and effective in humans thus far and continues to pique interest as an alternative therapy option [7, 8]. The anti-inflammatory properties of this treatment were exemplified, as our patient had no evidence of inflammation notable on T2-weighted MRI five weeks postoperatively (Fig. 3b). Given the positive results, we suggest continued research into the beneficial properties of amniotic membrane patches in cardiac surgery.

## Conclusion

Constrictive pericarditis is difficult to treat, and even a pericardiectomy may not offer a cure or desired long-term result. Given the inflammatory processes central to this disease process, amniotic membrane patches were used as anti-inflammatory treatment in a patient with a complicated history. With the amniotic membrane patch treatment and pericardiectomy, our patient had no evidence of inflammation five weeks postoperatively on T2-weighted MRI, highlighting the importance of continued research in this area.

## Additional file

Additional file 1: CARE Checklist – 2016: Information for writing a case report. (DOCX 603 kb)

## Abbreviations

BID: Bis in die (twice a day)
JVP: Jugular venous pressure
MRI: Magnetic resonance imaging
pAMR: Pathologic antibody-mediated rejection
