# Supplementary material for: Anti-inflammatory properties of amniotic membrane patch following pericardiectomy for constrictive pericarditis
Source: J Cardiothorac Surg. 2017 Jan 26;12:6. doi: 10.1186/s13019-017-0567-7 (PMC5270242; doi:10.1186/s13019-017-0567-7)
Supplement: Additional file 1: — CARE Checklist – 2016: Information for writing a case report. (DOCX 603 kb) [file 13019_2017_567_MOESM1_ESM.docx]

**CARE Checklist – 2016: Information for writing a case report**

**Topic Item Checklist item description Line/Page**

**Title 1** The words “case report” should be in the title along with the area of focus ___1___

**Key Words 2** Four to seven key words—include “case report” as one of the key words ___1___

**Abstract 3a** Background: What does this case report add to the medical literature? ___2___

**3b** Case summary: chief complaint, diagnoses, interventions, and outcomes ___2___

**3c** Conclusion: What is the main “take-away” lesson from this case? ___2___

**Introduction 4** The current standard of care and contributions of this case—with references (1-2 paragraphs) ___3___

**Timeline 5** Information from this case report organized into a timeline (table or figure) __N/A__

**Patient Information 6a** De-identified demographic and other patient or client specific information ___4___

**6b** Chief complaint—what prompted this visit? ___4___

**6c** Relevant history including past interventions and outcomes ___4___

**Physical Exam 7** Relevant physical examination findings ___4___

**Diagnostic 8a** Evaluations such as surveys, laboratory testing, imaging, etc. ___4___

**Assessment 8b** Diagnostic reasoning including other diagnoses considered and challenges ___4___

**8c** Consider tables or figures linking assessment, diagnoses and interventions ___5___

**8d** Prognostic characteristics where applicable ___4___

**Interventions 9a** Types such as life-style recommendations, treatments, medications, surgery ___5___

**9b** Intervention administration such as dosage, frequency and duration ___5___

**9c** Note changes in intervention with explanation ___5___

**9d** Other concurrent interventions ___5___

**Follow-up and 10a** Clinician assessment (and patient or client assessed outcomes when appropriate) ___5___

**Outcomes 10b** Important follow-up diagnostic evaluations ___5___

**10c** Assessment of intervention adherence and tolerability, including adverse events __ 5 _ _

**Discussion 11a** Strengths and limitations in your approach to this case __ 6 _

**11b** Specify how this case report informs practice or Clinical Practice Guidelines (CPG) ___ 6 _

**11c** How does this case report suggest a testable hypothesis? __6 & 7_

**11d** Conclusions and rationale ___ 7___

**Patient Perspective 12** When appropriate include the assessment of the patient or client on this episode of care __N/A__

**Informed Consent 13** Informed consent from the person who is the subject of this case report is required by most journals __N/A _

**Additional Information 14** Acknowledgement section; Competing Interests; IRB approval when required ___1___
